# Tissue MicroRNAs in Arrhythmogenic Cardiomyopathy: A Systematic Review of Studies in Human Myocardium and Animal Models with Implications for Post-Mortem Molecular Diagnostics

**DOI:** 10.3390/genes17060725

**Published:** 2026-06-22

**Authors:** Gabriele Napoletano, Alessandro Ghamlouch, Maura Racciatti, Elena Sonnini, Biancamaria Treves, Gaia De Angelis, Filippo Alessandro Montalto, Aniello Maiese, Raffaele La Russa, Paola Frati, Alessandra De Matteis

**Affiliations:** 1 Department of Anatomical, Histological, Forensic and Orthopedic Sciences, Sapienza University of Rome, 00161 Rome, Italy; 2 Medicina Genomica, Dipartimento Scienze Della Vita e Sanità Pubblica, Università Cattolica del Sacro Cuore, 00168 Rome, Italy; 3 Department of Clinical Medicine, Public Health, Life Sciences, and Environmental Sciences, University of L’Aquila, 67100 L’Aquila, Italy; 4 ASL Salerno—UOC Medicina Legale ed Assicurativa, 84078 Vallo della Lucania, SA, Italy

**Keywords:** arrhythmogenic cardiomyopathy (ARVC), microRNAs (miRNA), myocardial fibrosis, desmosomal dysfunction, cardiac remodeling

## Abstract

Arrhythmogenic cardiomyopathy (ACM/ARVC) is an inherited myocardial disease characterized by progressive fibro-fatty replacement, ventricular arrhythmias, and an increased risk of sudden cardiac death. In addition to mutations in desmosomal genes, growing evidence suggests that microRNAs (miRNAs) actively contribute to disease pathogenesis by regulating key processes such as fibrosis, cell adhesion, and cardiac remodeling. This systematic review analyzed the main miRNAs identified in studies of human cardiac tissue and animal models of ARVC. Materials and Methods: Studies based on human myocardial tissue analysis (including autopsy and biopsy samples) and animal models of arrhythmogenic cardiomyopathy were included, using RNA sequencing, small RNA sequencing, miRNA arrays, and RT-qPCR. Studies on circulating miRNAs and narrative reviews were excluded. miRNAs were analyzed in relation to their functional pathways and their role in disease pathogenesis. Results: The synthesis of studies on human and animal cardiac tissue reveals a consistent miRNA signature associated with arrhythmogenic cardiomyopathy. MiR-21-5p and miR-29b-3p are associated with fibrosis and extracellular matrix remodeling, whereas miR-133a-b and miR-130a are linked to cardiomyocyte integrity loss and desmosomal dysfunction. A second group of miRNAs, including miR-217-5p, miR-708-5p, and miR-135b, regulates key pathways such as Wnt/β-catenin and Hippo signaling, contributing to structural remodeling and loss of cellular identity. Furthermore, downregulation of miR-499-5p is associated with mitochondrial dysfunction and cellular vulnerability, while the miR-142-3p, miR-182-5p, and miR-183-5p clusters contribute to differential molecular signatures compared with other cardiomyopathies. Overall, miRNAs converge on three main pathogenic axes: myocardial fibrosis, desmosomal impairment, and remodeling of cellular signaling pathways. Conclusions: The available evidence indicates that arrhythmogenic cardiomyopathy is regulated by a coordinated network of miRNAs that actively drives myocardial damage progression. These miRNAs represent not only biomarkers but also functional mediators of disease, suggesting potential diagnostic and therapeutic applications based on tissue-specific molecular signatures, including in post-mortem settings.

## 1. Introduction

Arrhythmogenic cardiomyopathy (ACM) is an inherited myocardial disease, typically transmitted as an autosomal dominant trait, characterized by ventricular electrical instability, progressive loss of cardiomyocytes, and fibro-fatty replacement of the myocardium. Its estimated prevalence ranges from approximately 1:2000 to 1:5000 individuals, and it is particularly relevant in young subjects and athletes because of the increased risk of sudden cardiac death [1,2,3,4]. Historically described as a disease predominantly affecting the right ventricle, ACM is now recognized to encompass right-dominant, biventricular, and left-dominant phenotypes. From a pathological perspective, fibro-fatty replacement may initially develop at the subepicardial level and involve regions classically included in the so-called “triangle of dysplasia”, subsequently extending toward the endocardium and, in more advanced stages, involving the left ventricle as well [2,5]. Clinical manifestations include ventricular premature beats, ventricular tachycardia, palpitations, syncope, heart failure, and sudden cardiac death [6,7,8]. The genetic basis of the disease is frequently represented by variants in genes encoding desmosomal proteins, including *PKP2*, *DSP*, *DSG2*, *DSC2*, and *JUP*, although non-desmosomal genes have also been described. Diagnosis remains multiparametric and integrates clinical, electrocardiographic, arrhythmic, morpho-functional, histopathological, and genetic data, according to the Task Force Criteria and the more recent Padua criteria [5,7,9].

However, incomplete penetrance, phenotypic variability, and gene-elusive forms suggest that genetic mutation alone is insufficient to explain the entire clinical spectrum of the disease [3,10]. ACM-associated variants impair not only the mechanical adhesion between cardiomyocytes but also intracellular signaling pathways. In particular, suppression of the Wnt/β-catenin pathway and activation of the Hippo pathway have been associated with adipogenesis, fibrosis, apoptosis, and disruption of the intercalated discs [10]. These processes contribute to the progressive thinning of the myocardial wall, contractile dysfunction, and electrical instability, predisposing to ventricular arrhythmias [2,7]. For a long time, ACM was predominantly interpreted as a disease of cardiomyocytes. More recent evidence, however, indicates a significant involvement of the cardiac microenvironment, including cardiac stromal cells, fibroblasts, and immune cells [10]. Cardiac stromal cells derived from patients with ACM express desmosomal proteins and exhibit an increased propensity toward adipogenic differentiation, suggesting that fibro-fatty remodeling arises from a multicellular network more complex than myocardial damage alone [10,11,12,13]. In this context, microRNAs (miRNAs) represent a regulatory layer of particular interest. These are small non-coding RNAs, approximately 19–25 nucleotides in length, capable of modulating gene expression through binding to target transcripts, promoting mRNA degradation, or inducing translational inhibition [10]. In the cardiovascular context, they participate in cellular differentiation, fibrosis, inflammation, cell death, electrical homeostasis, and structural remodeling [14]. In ACM, their relevance is twofold: they may contribute to disease pathogenesis by regulating critical signaling pathways and, at the same time, serve as measurable biomarkers in tissue, blood, or post-mortem samples. This study aims to systematically synthesize investigations that have analyzed miRNAs in human myocardial tissue, autopsy or biopsy samples, and animal models of arrhythmogenic cardiomyopathy, with particular attention to their diagnostic implications and potential applications in post-mortem molecular diagnostics.

## 2. Materials and Methods

**Figure 1:**
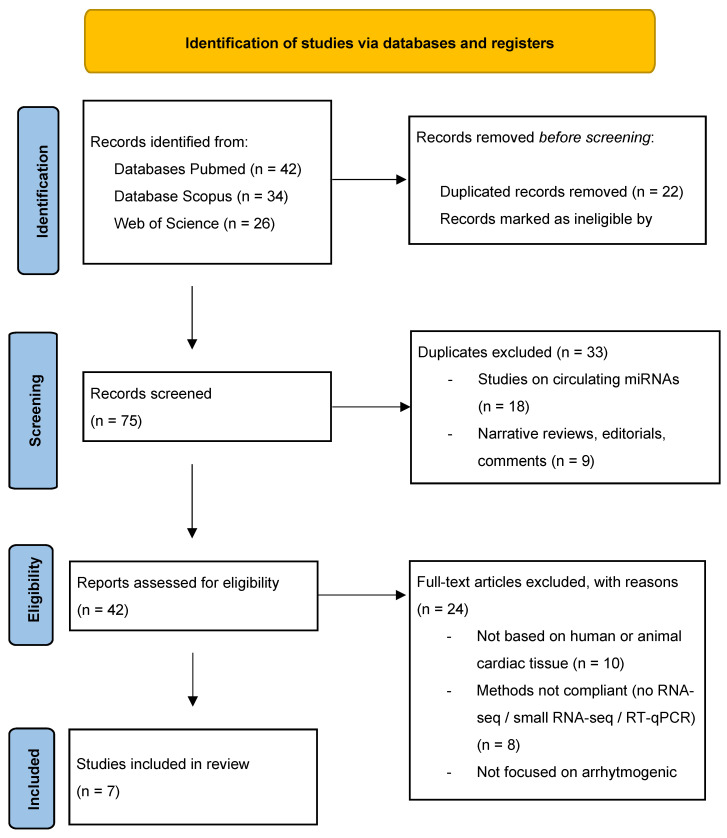
The selection of papers following the PRISMA protocol.

The review was conducted in accordance with the PRISMA 2020 recommendations for reporting systematic reviews [15]. Original studies analyzing miRNAs in human myocardial tissue, including autopsy and biopsy samples, or in experimental animal models of ACM, were included. Studies based on gene expression profiling methodologies such as RNA sequencing, small RNA sequencing, miRNA arrays, and RT-qPCR were considered eligible (Figure 1). Studies based exclusively on circulating miRNAs, narrative reviews, editorials, letters, case reports, and works lacking original data on cardiac tissue or relevant experimental models were excluded. The bibliographic search was conducted in electronic databases including PubMed/MEDLINE, Scopus, and Web of Science, using combinations of keywords related to “arrhythmogenic cardiomyopathy”, “arrhythmogenic right ventricular cardiomyopathy”, “microRNA”, “miRNA”, “cardiac tissue”, “myocardium”, “animal model”, “RNA sequencing”, and “RT-qPCR.” Two independent reviewers performed title/abstract and full-text screening, with discrepancies resolved by consensus. Extracted data included author and year, experimental model, sample type, analytical technique, identified miRNAs, direction of expression, proposed pathways or targets, and interpretative relevance. The studies included in Table 1 represent the main original works analyzing miRNAs in human myocardial tissue, patient-derived cardiac cells, autopsy samples, experimental models, or integrated lncRNA–miRNA–mRNA regulatory networks in arrhythmogenic cardiomyopathy. In particular, the contributions of Zhang et al. [14], Mazurek et al. [16], Rainer et al. [17], Calore et al. [18], Bueno Marinas et al. [19], Bonet et al. [20], and Li et al. [13]. were included, as they are directly focused on the role of miRNAs and post-transcriptional regulation in ACM/ARVC.

### Quality Assessment

Given the translational nature of the included studies, which comprised analyses of human myocardial tissue, cardiac stromal cells, animal models, and transcriptomic datasets, conventional risk-of-bias tools designed for randomized clinical trials or observational epidemiological studies were considered inappropriate. Therefore, a domain-based methodological quality assessment was performed (Table 2). Each study was evaluated according to the following domains: adequacy of arrhythmogenic cardiomyopathy diagnosis and case characterization; appropriateness of control selection; sample size and representativeness; methodological description of miRNA extraction, quantification, and normalization procedures; appropriateness of statistical analyses, including correction for multiple testing where applicable; validation of findings in independent cohorts, tissues, or experimental models; and completeness of outcome reporting. For each domain, studies were classified as presenting low, moderate, or high risk of methodological bias. An overall quality judgment (high, moderate, or low quality) was subsequently assigned based on the collective assessment of all domains. Attention was given to sample size, tissue source, and independent validation, as these represent major sources of variability in myocardial miRNA research.

## 3. Results

The synthesis of the included studies highlights a miRNA signature associated with arrhythmogenic cardiomyopathy, although with substantial heterogeneity across experimental models, sample types, and analytical platforms. When findings are interpreted according to biological themes, partially overlapping patterns emerge, with both consistent and contrasting results across studies [14,16,17,19,20].

### 3.1. Signaling Pathway-Related miRNAs (Wnt/Hippo)

With regard to miRNAs associated with signaling pathways (Wnt/Hippo), Zhang et al. identified in human right ventricular myocardial samples 21 validated differentially expressed miRNAs, including upregulated miR-21-5p and downregulated miR-135b, associated with Wnt/Hippo signaling as well as myocardial adipogenesis and fibrosis [14]. Consistently, in the DSG2 p.Q558* transgenic murine model, Calore et al. identified 24 deregulated miRNAs, including upregulated miR-217-5p and miR-708-5p and downregulated miR-499-5p, linked to suppression of the Wnt/β-catenin pathway, an early event potentially involved in fibro-adipogenic transition [18]. Although the specific miRNAs differ between studies, a consistent finding is the involvement of Wnt-related pathways, while the variability in individual miRNA profiles represents a point of divergence.

### 3.2. Desmosome-Related miRNAs

With regard to miRNAs associated with desmosomal integrity, the functional role of miRNAs emerges particularly clearly in the αMHC-miR-130a murine model. In this model, cardiac overexpression of miR-130a directly represses DSC2 and induces an arrhythmogenic cardiomyopathy (ACM)-like phenotype characterized by right ventricular dilation, ventricular premature beats, fibrosis, and lipid accumulation [16]. This finding provides consistent mechanistic evidence linking miRNA dysregulation to desmosomal impairment. However, a contrasting element is that this evidence is primarily derived from animal models and has not been consistently confirmed in human studies.

### 3.3. Fibrosis-Related miRNAs

With regard to miRNAs associated with fibrosis and extracellular matrix remodeling, Rainer et al. demonstrated increased expression of miR-520c-3p, miR-29b-3p, and miR-1183 in cardiac stromal cells, associated with extracellular matrix organization, collagen regulation, and cell adhesion [17]. These findings are consistent with those of Bonet et al., who identified enrichment in pathways related to extracellular matrix organization, inflammation, and apoptosis, supported by negatively correlated miRNA–mRNA pairs. Thus, fibrotic and extracellular matrix pathways represent a coherent and reproducible biological signal across studies. Nevertheless, the specific miRNAs involved are not identical, highlighting inter-study variability [20].

Finally, the comparison between tissue and circulating miRNAs further underscores both consistency and discrepancy. Bueno Marinas et al. identified a circulating panel of six miRNAs with potential biomarker value (miR-122-5p, miR-133a-3p, miR-133b, miR-142-3p, miR-182-5p, and miR-183-5p). However, the tissue–blood comparison suggests that circulating miRNA profiles only partially reflect myocardial remodeling. This represents a consistent limitation across studies, indicating that circulating miRNAs capture both cardiac-specific and systemic responses, thereby contributing to heterogeneity [19].

Overall, the studies converge on key pathogenic processes, including Wnt/Hippo signaling, desmosomal integrity, and fibrotic remodeling, while diverging in the specific miRNAs identified, likely reflecting differences in experimental design, biological samples, and disease stages.

## 4. Discussion

Overall, the available data support a network-based view of ACM, in which genetics, cell adhesion, intracellular signaling, stromal remodeling, inflammation, and post-transcriptional regulation interact dynamically. miRNAs do not appear to be mere epiphenomena of myocardial injury, but rather potential intermediate regulators of central processes such as fibrosis, adipogenesis, apoptosis, mitochondrial dysfunction, and intercalated disc disruption. In the forensic setting, post-mortem cardiac investigation requires an integrated approach capable of distinguishing structural pathology, trauma, genetic alterations, and potential arrhythmic substrates [4,21,22]. The first pathogenic axis involves fibrosis and extracellular matrix remodeling. miR-21-5p and miR-29b-3p emerge as relevant candidates due to their association with fibrosis, collagen regulation, and stromal responses [14,17]. In human samples, fibrosis does not appear to be merely a terminal consequence of myocyte loss, but rather a structural component of the pathological phenotype, sustained by interactions among cardiomyocytes, fibroblasts, and stromal cells [17,20].

A second axis involves desmosomal integrity. The miR-130a model demonstrates that modulation of DSC2 can lead to desmosome disruption and the emergence of an ACM-like phenotype [16]. This finding is particularly important because it directly links miRNA-mediated regulation to a classical structural gene of the disease, suggesting that the reduction in desmosomal proteins may arise not only from genetic variants but also from post-transcriptional mechanisms. A third axis involves the Wnt/β-catenin and Hippo signaling pathways. Suppression of Wnt signaling, observed both in experimental models and human samples, represents a key event in the transition toward a fibro-adipogenic phenotype [10,14,18]. miRNAs appear to act as intermediate regulators of this suppression, modulating both upstream and downstream components of the pathway. In parallel, dysregulation of the Hippo pathway contributes to loss of tissue homeostasis, apoptosis, and activation of maladaptive transcriptional programs [14]. Integrated miRNA–mRNA analyses show that miRNAs operate within highly interconnected networks: a single miRNA can modulate multiple targets, and the same gene can be regulated by several miRNAs. This regulatory redundancy may help explain the phenotypic heterogeneity of ACM even in the presence of similar genetic variants [20]. This perspective is consistent with the clinical variability of the disease, which includes right-dominant, biventricular, and left-dominant phenotypes [2,4,5]. From a translational perspective, circulating miRNAs offer important theoretical advantages: they are accessible through minimally invasive methods, potentially repeatable over time, and can be integrated with clinical, genetic, and imaging data [10,19]. However, their biological significance remains complex. Some circulating miRNAs may reflect myocardial injury, fibrosis, or inflammation, whereas others originate from immune cells, stromal cells, or systemic responses to cardiac stress [10,19]. For this reason, circulating miRNAs should be interpreted as integrative biomarkers rather than unambiguous indicators of a single pathological process. From the perspective of post-mortem molecular diagnostics, tissue miRNAs have specific potential. Analysis of autopsy samples could integrate histology, genetics, and immunohistochemistry in cases of sudden death with suspected ACM, particularly when morphological alterations are early, focal, or inconclusive [4,20]. The work by Li et al. further extends this framework, extending the analysis from single miRNA deregulation to the construction of an lncRNA–miRNA–mRNA ceRNA network in ARVC. The study identified hub genes and lncRNAs, including *COL1A1*, *COL5A1*, *FBN1*, *BGN*, *XIST*, and *LINC00173*, primarily associated with extracellular matrix organization, collagen deposition, and fibrosis, thereby reinforcing the hypothesis that non-coding RNA regulation contributes to structural remodeling in the disease [13]. However, forensic applications require rigorous standardization of sampling procedures, post-mortem interval, tissue preservation, RNA extraction methods, normalization strategies, and interpretative thresholds.

## 5. Limitations

The available literature presents several limitations. The first is methodological heterogeneity: studies differ in sample type, collection and storage procedures, analytical platforms, data normalization, and statistical strategies. This makes direct comparison of results difficult and may explain the limited convergence among proposed miRNA signatures. In addition, most studies are characterized by small sample sizes (often *n* ≤ 4–24), which reduces statistical power and further limits the reproducibility and generalizability of findings.

The second limitation is the clinical heterogeneity of the cohorts. The ACM label encompasses right-dominant, biventricular, and left-dominant phenotypes; patients with known mutations and gene-elusive cases; and those at early and advanced disease stages. It is plausible that different subtypes exhibit distinct miRNA profiles and that the lack of fine phenotyping reduces the ability to identify reproducible signals.

The third limitation is the scarcity of longitudinal studies. Most data are cross-sectional and allow association of a given miRNA with disease presence, but do not define its prognostic value or its ability to monitor disease progression, arrhythmic burden, or therapeutic response.

The fourth limitation concerns pre-analytical variables, particularly in studies based on autopsy material. Factors such as post-mortem interval (PMI) and tissue handling may significantly influence miRNA stability. RNA degradation represents a relevant issue in post-mortem samples and may affect the reliability of expression profiles, which is particularly critical in forensic applications.

## 6. Conclusions

The available evidence indicates that miRNAs play a relevant role in arrhythmogenic cardiomyopathy, both as biological modulators of crucial signaling pathways and as potential biomarkers. Studies in cardiac tissue and animal models suggest the involvement of miRNAs in the regulation of desmosomal integrity, Wnt/β-catenin and Hippo pathways, as well as fibrosis, adipogenesis, inflammation, and mitochondrial dysfunction. Despite these promising findings, the translation of miRNAs into clinically or forensically reliable biomarkers for ACM remains incomplete. The main barriers include methodological heterogeneity, limited cohort sizes, lack of longitudinal studies, and insufficient functional validation of many candidate miRNAs. The future of the field will depend on multicenter, standardized studies capable of integrating genomics, epigenomics, transcriptomics, imaging, clinical phenotyping, and post-mortem data. In summary, miRNAs do not appear to be mere bystanders of the disease: for several of them, there is compelling evidence suggesting an active role in ACM pathogenesis. However, before they can be considered mature tools for clinical practice or post-mortem molecular diagnostics, broader, prospective, and functional validation is still required.

## Figures and Tables

**Table 1:** Studies on tissue miRNAs or using experimental models of arrhythmogenic cardiomyopathy.

Author (Year)	Model/Sample	Technique	miRNAs Identified	Expression Change #	Target/Pathway	Note
Zhang et al. (2016) [14]	Human, right ventricular myocardium; 24 ARVC hearts from transplantation vs. 24 autopsy/donor controls	S-Poly(T) Plus qRT-PCR on 1078 miRNAs, with individual validation	21 validated miRNAs; relevant: miR-21-5p, miR-135b, miR-34a-5p, miR-212-3p	11 ↑. 10 ↓; miR-21-5p ↑. miR-135b ↓	Wnt-Hippo; association with adipose and myocardial fibrosis	First extended profiling on ARVC human cardiac tissue [9].
Mazurek * et al. (2017) [16]	aMHC-miR-130a transgenic mouse model; ventricular myocardium of miR-130a inducible mice vs. controls	Phenotyping, Western blot, immunofluorescence, histology, luciferase assay	miR-130a	↑	Direct repression of DSC2; Desmosome—intercalary disc	Overexpression of miR-130a induces an ACM-like phenotype with right ventricular dilation, PVC, fibrosis, and lipid accumulation [7].
Rainer et al. (2018) [17]	Human, cardiac stromal cells from endomyocardial biopsy; profiling: 3 ACM vs. 3 controls; validation: 8 ACM vs. 5 controls	TaqMan Low-Density Array, RNA-seq, RT-qPCR	miR-520c-3p, miR-29b-3p, miR-1183	All ↑ (>4x)	Cell adhesion, ECM, collagen, ephrin signaling	Important study because it shifts the focus from the cardiomyocyte to the stromal-fibrotic compartment [10].
Calore et al. (2019) [18]	Transgenic murine cardiac model carrying DSG2 p.Q558; Tg-hQ hearts vs. non-transgenic hearts.	RNA-seq miRNA + bioinformatic	24 deregulated miRNAs; Main: miR-217-5p, miR-708-5p, miR-499-5p	miR-217-5p ↑, miR-708-5p ↑, miR-499-5p #x2193;	Wnt/ß-catenin	Strong evidence of Wnt suppression and early miRNA signature in a desmosomal model [11].
Bueno Marinas et al. (2020) [19]	Human, right ventricle + peripheral blood; discovery: 9 RV ACM tissues, 9 ACM blood samples, 4 controls; validation on 90 ACM	84-miRNA array, NGS/small RNA-seq, qPCR	10 miRNAs shared tissue-blood; validated panel: miR-122-5p, miR-133a-3p, miR-133b, miR-142-3p, miR-182-5p, miR-183-5p	Mixed profile; Some directions diverge between tissue and blood	Pathway ACM-correlated; genotype-related and Hippo signaling	Bridging study between tissue and circulating biomarkers; useful, but only a part of the circulating signal directly reflects the myocardium [12].
Bonet et al. (2024) [20]	Human, autopsy hearts; 4 ACM frozen RV biopsies vs. 4 post-mortem controls	mRNA-seq + small RNA-seq + interactome miRNA-mRNA	8 miRNA DE: miR-135a-5p, miR-140-3p, miR-145-5p, miR-486-5p, miR-486-3p, miR-125a-5p, let-7e-5p, let-7d-3p	3 ↑, 5 ↓	Mitochondrion/respiration, oxidative stress, apoptosis, inflammation, ECM	First integrated miRNA-mRNA study on autopsy hearts ACM; very relevant for forensic medicine [13].
Li et al. (2024) [13]	ARVC human datasets and non-failing controls from GEO; validation of mRNA and IncRNA in human cardiac tissue	Bioinformatics analysis of mRNA/IncRNA datasets, DEmiR from previous profiling, target prediction with starBase, IncRNA-miRNA-mRNA | ceRNA network, PPI, WGCNA, RT-qPCR	DEmiRs identified from previous protiling studies; ceRNA network focused on molecular interactions IncRNA-miRNA-mRNA	Integrated differential expression of DEM, DEInc and DEmiR	ECM, fibrosis, organization of the extracellular matrix, collagen; hubs: COL1A1, COL5A1, FBN1, BGN, XIST, LINC00173.	Bioinformatic-integrative study proposing an IncRNA-miRNA-mRNA regulatory network and a predictive diagnostic model for ARVC; useful for linking miRNA, IncRNA and fibrotic remodeling.

* Mazurek et al. (2017) [16] is presented as an example of mechanistic research relevant to the discussed biological pathways. This study was not included in the systematic review quality assessment and is therefore not included in Table 1. # Upward (↑) and downward (↓) arrows indicate upregulation and downregulation of RNA expression, respectively.

**Table 2:** Quality assessment.

Study	Human Tissue/Animal Model	Case Definition	Controls	Sample Size	Molecular Methods	Validation	Overall Quality
Zhang et al. (2016) [14]	Human myocardium	Low risk	Low risk	Moderate risk	Low risk	Low risk	High
Rainer et al. (2018) [17]	Human cardiac stromal cells	Low risk	Low risk	Moderate risk	Low risk	Moderate risk	High
Calore et al. (2019) [18]	Murine ACM model	Low risk	Low risk	Moderate risk	Low risk	Low risk	High
Bueno Ma-rinas et al. (2020) [19]	Human ACM cohort	Low risk	Low risk	Low risk	Low risk	Moderate risk	High
Bonet et al. (2024) [20]	Human post-mortem myocardium	Low risk	Low risk	High risk	Low risk	Moderate risk	Moderate
Li et al. (2024) [13]	Human tissue + bioinformatic datasets	Low risk	Moderate risk	Moderate risk	Low risk	Moderate risk	Moderate
Mazurek * et al. (2017) [16]	Narrative review	Not applicable	Not applicable	Not applicable	Not applicable	Not applicable	Excluded

* Mazurek et al. (2017) [16] is presented as an example of mechanistic research relevant to the discussed biological pathways. This study was not included in the systematic review quality assessment.

## Data Availability

No new data were created or analyzed in this study.

## References

[1] de la Guía-Galipienso F., Ugedo-Alzaga K., Grazioli G., Quesada-Ocete F.J., Feliu-Rey E., Perez M.V., Quesada-Dorador A., Sanchis-Gomar F. (2023). Arrhythmogenic Cardiomyopathy and Athletes: A Dangerous Relationship. Curr. Probl. Cardiol..

[2] Corrado D., Link M.S., Calkins H. (2017). Arrhythmogenic Right Ventricular Cardiomyopathy. N. Engl. J. Med..

[3] Basso C., Corrado D., Marcus F.I., Nava A., Thiene G. (2009). Arrhythmogenic right ventricular cardiomyopathy. Lancet.

[4] Duca F.D., Ghamlouch A., Manetti A.C., Napoletano G., Sonnini E., Treves B., De Matteis A., La Russa R., Sheppard M.N., Fineschi V. (2024). Sudden Cardiac Death, Post-Mortem Investigation: A Proposing Panel of First Line and Second Line Genetic Tests. J. Pers. Med..

[5] Corrado D., Marra M.P., Zorzi A., Beffagna G., Cipriani A., Lazzari M., Migliore F., Pilichou K., Rampazzo A., Rigato I. (2020). Diagnosis of arrhythmogenic cardiomyopathy: The Padua criteria. Int. J. Cardiol..

[6] Towbin J.A., McKenna W.J., Abrams D.J., Ackerman M.J., Calkins H., Darrieux F.C., Daubert J.P., de Chillou C., DePasquale E.C., Desai M.Y. (2019). 2019 HRS expert consensus statement on evaluation, risk stratification, and management of arrhythmogenic cardiomyopathy. Heart Rhythm.

[7] Marcus F.I., McKenna W.J., Sherrill D., Basso C., Bauce B., Bluemke D.A., Calkins H., Corrado D., Cox M.G., Daubert J.P. (2010). Diagnosis of arrhythmogenic right ventricular cardiomyopathy/dysplasia: Proposed Modification of the Task Force Criteria. Eur. Heart J..

[8] Magon F., Longhitano Y., Savioli G., Piccioni A., Tesauro M., Del Duca F., Napoletano G., Volonnino G., Maiese A., La Russa R. (2024). Point-of-Care Ultrasound (POCUS) in Adult Cardiac Arrest: Clinical Review. Diagnostics.

[9] Zaghlol R., Dey A.K., Barac A. (2020). Takotsubo and cancer: Takotsubo cardiomyopathy in the era of emerging cancer therapies. Eur. Heart J..

[10] Bueno Marinas M., Celeghin R., Cason M., Thiene G., Basso C., Pilichou K. (2020). The Role of MicroRNAs in Arrhythmogenic Cardiomyopathy: Biomarkers or Innocent Bystanders of Disease Progression?. Int. J. Mol. Sci..

[11] Garcia-Gras E., Lombardi R., Giocondo M.J., Willerson J.T., Schneider M.D., Khoury D.S., Marian A.J. (2006). Suppression of canonical Wnt/β-catenin signaling by nuclear plakoglobin recapitulates phenotype of arrhythmogenic right ventricular cardiomyopathy. J. Clin. Investig..

[12] O’Neill K.M., Campbell D.C., Edgar K.S., Gill E.K., Moez A., McLoughlin K.J., O’Neill C.L., Dellett M., Hargey C.J., Abudalo R.A. (2020). NOX4 is a major regulator of cord blood-derived endothelial colony-forming cells which promotes post-ischaemic revascularization. Cardiovasc. Res..

[13] Li H., Song S., Shi A., Hu S. (2024). Identification of Potential lncRNA-miRNA-mRNA Regulatory Network Contributing to Arrhythmogenic Right Ventricular Cardiomyopathy. J. Cardiovasc. Dev. Dis..

[14] Zhang H., Liu S., Dong T., Yang J., Xie Y., Wu Y., Kang K., Hu S., Gou D., Wei Y. (2016). Profiling of differentially expressed microRNAs in arrhythmogenic right ventricular cardiomyopathy. Sci. Rep..

[15] Page M.J., McKenzie J.E., Bossuyt P.M., Boutron I., Hoffmann T.C., Mulrow C.D., Shamseer L., Tetzlaff J.M., Akl E.A., Brennan S.E. (2021). The PRISMA 2020 statement: An updated guideline for reporting systematic reviews. BMJ.

[16] Mazurek S.R., Calway T., Harmon C., Farrell P., Kim G.H. (2017). MicroRNA-130a Regulation of Desmocollin 2 in a Novel Model of Arrhythmogenic Cardiomyopathy. Microrna.

[17] Rainer J., Meraviglia V., Blankenburg H., Piubelli C., Pramstaller P.P., Paolin A., Cogliati E., Pompilio G., Sommariva E., Domingues F.S. (2018). The arrhythmogenic cardiomyopathy-specific coding and non-coding transcriptome in human cardiac stromal cells. BMC Genom..

[18] Calore M., Lorenzon A., Vitiello L., Poloni G., Khan M.A.F., Beffagna G., Dazzo E., Sacchetto C., Polishchuk R., Sabatelli P. (2019). A novel murine model for arrhythmogenic cardiomyopathy points to a pathogenic role of Wnt signalling and miRNA dysregulation. Cardiovasc. Res..

[19] Marinas M.B., Celeghin R., Cason M., Bariani R., Frigo A.C., Jager J., Syrris P., Elliott P.M., Bauce B., Thiene G. (2020). A microRNA Expression Profile as Non-Invasive Biomarker in a Large Arrhythmogenic Cardiomyopathy Cohort. Int. J. Mol. Sci..

[20] Bonet F., Campuzano O., Córdoba-Caballero J., Alcalde M., Sarquella-Brugada G., Braza-Boïls A., Brugada R., Hernández-Torres F., Quezada-Feijoo M., Ramos M. (2024). Role of miRNA–mRNA Interactome in Pathophysiology of Arrhythmogenic Cardiomyopathy. Biomedicines.

[21] Treves B., Sonnini E., Russa R.L., Del Duca F., Ghamlouch A., De Matteis A., Trignano C., Marchal J.A., Carrillo E., Napoletano G. (2024). Can Hemorrhagic Stroke Genetics Help Forensic Diagnosis in Pediatric Age (<5 Years Old)?. Genes.

[22] Napoletano G., Treves B., Paola L.D., Del Duca F., Ghamlouch A., Frati P., Maiese A. (2024). Impact of Cardiac Surgery Scar on Heart Rupture Following a Fall from Height. Diagnostics.

